# Severe hypotension following sublingual dexmedetomidine administration in an elderly patient

**DOI:** 10.1093/ajhp/zxaf335

**Published:** 2025-12-06

**Authors:** Zlatan Coralic, Moujan Allai, Calvin Hwang, Benjamin Michaels, Paul Takamoto

**Affiliations:** Department of Clinical Pharmacy and Emergency Medicine, University of California San Francisco, San Francisco, CA, USA; University of California San Francisco School of Pharmacy, San Francisco, CA, USA; Department of Clinical Pharmacy, University of California San Francisco, San Francisco, CA, USA; Department of Clinical Pharmacy, University of California San Francisco, San Francisco, CA, USA; Department of Clinical Pharmacy, University of California San Francisco, San Francisco, CA, USA

**Keywords:** alpha-2 agonist, causality assessment, Naranjo criteria, pharmacovigilance, sublingual dexmedetomidine

## Abstract

**Purpose:**

Sublingual dexmedetomidine is a recently introduced α_2_-adrenergic agonist approved for management of agitation in patients with schizophrenia or bipolar disorder. Published safety data on the use of this drug outside of clinical trials is limited.

**Summary:**

We describe an 88-year-old patient with hypertension, vascular dementia, and non–ST segment elevation myocardial infarction who experienced profound hypotension after receiving sublingual dexmedetomidine for agitation. The patient was boarding in the emergency department due to hospital overcrowding and developed delirium with severe agitation. After QT interval prolongation limited further antipsychotic use, a 120-µg dose of sublingual dexmedetomidine was administered. Within 1 hour, the patient’s blood pressure fell from 102/49 to 62/40 mm Hg. Concomitantly, her hemoglobin declined from 12.1 to 6.9 g/dL, prompting a blood transfusion and investigation of active bleeding. Imaging revealed a large intramuscular hematoma possibly related to intramuscular injections in the setting of therapeutic anticoagulation and dual antiplatelet therapy. The Naranjo score was 4, indicating a possible adverse drug reaction to dexmedetomidine; however, the strong temporal association with sublingual administration supports a contributory role.

**Conclusion:**

This case highlights the hemodynamic vulnerability of older adults, especially those with comorbidities and recent anticoagulation. While sublingual dexmedetomidine offers a noninvasive option for agitation management, its hypotensive potential may be clinically significant in this population. Our findings underscore the need for careful patient selection, dose optimization, and close monitoring when using dexmedetomidine in elderly patients, especially those with limited cardiovascular reserve. Further research is needed to guide safe dosing in geriatric and medically complex populations.

Dexmedetomidine is an α_2_-adrenergic agonist used for its sedative and anxiolytic properties.^1,2^ Initially approved in 1999, its intravenous formulation is commonly used for procedural sedation and in mechanically ventilated patients.^1^ A new sublingual formulation was approved in 2022 for the treatment of agitation associated with schizophrenia or bipolar I or II disorder.^2^ Although desired for its anxiolytic and sedative effects, dexmedetomidine can cause bradycardia and hypotension.^1,2^

In this report we present the case of an elderly patient who experienced profound hypotension after administration of sublingual dexmedetomidine.

## Case report

An 88-year-old female with a past medical history of hypertension, type 2 diabetes mellitus, and vascular dementia (previously treated with olanzapine) was brought into the emergency department (ED) by ambulance after an episode of dizziness and lightheadedness. Per family report, she had been experiencing poor oral intake, resulting in recent weight loss and overall decline. In the ED she endorsed mild shortness of breath but denied fever, cough, slurred speech, and chest pain. Her initial vital signs were as follows: blood pressure, 142/90 mm Hg; heart rate, 68 beats per minute; respiratory rate, 20 breaths per minute; temperature, 36.4 °C; and oxygen saturation, 99% on room air. The AWOL delirium score on arrival was 3, consistent with a high risk of the development of inpatient delirium. Aside from mild hypokalemia (serum potassium, 3.2 mEq/L), which was corrected with oral potassium supplementation, laboratory values were within normal limits, including liver function test results and magnesium levels. A chest radiograph, electrocardiogram, and noncontrast computed tomography (CT) scan of the brain were also conducted, with unremarkable findings. Serial troponin levels gradually increased from a baseline level of 0.02 ng/mL to 1.21 ng/mL. Treatment of suspected non–ST segment elevation myocardial infarction (NSTEMI) was initiated with a bolus of unfractionated heparin (60 units/kg), followed by an infusion at 12 units/kg/h, as well as dual antiplatelet therapy with clopidogrel 600 mg as a loading dose, followed by 75 mg daily; and aspirin 324 mg as a loading dose, followed by 81 mg daily. Patient disposition was admission to the hospital; however, due to hospital overcrowding and unavailability of inpatient beds, the patient continued to stay (ie, board) in a crowded ED.

On day 3, while still boarding in the ED, the patient became delirious and agitated, pulling at lines and attempting to leave the bed. As intravenous access became compromised, calming medications were given: intramuscular olanzapine 5 mg at 0134 hours (left thigh) and 0552 hours (left thigh), followed by intramuscular ziprasidone 10 mg at 0617 hours (right thigh) and 2307 hours (right deltoid).

**Figure 1.**
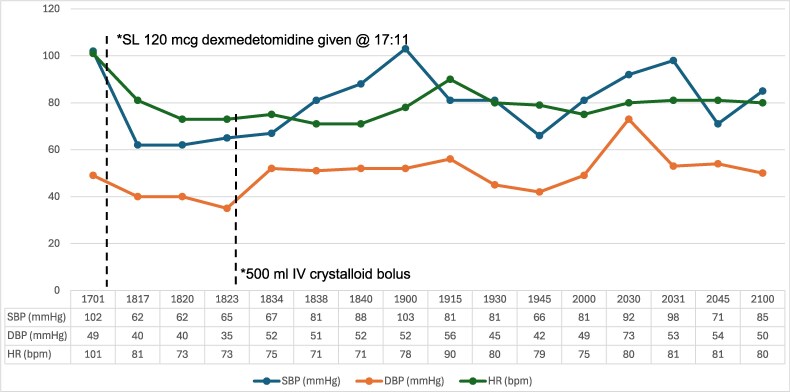
Blood pressure and heart rate (HR) trends after administration of 120 µg of sublingual (SL) dexmedetomidine to the patient. DBP indicates diastolic blood pressure; IV, intravenous; SBP, systolic blood pressure.

**Figure 2.**
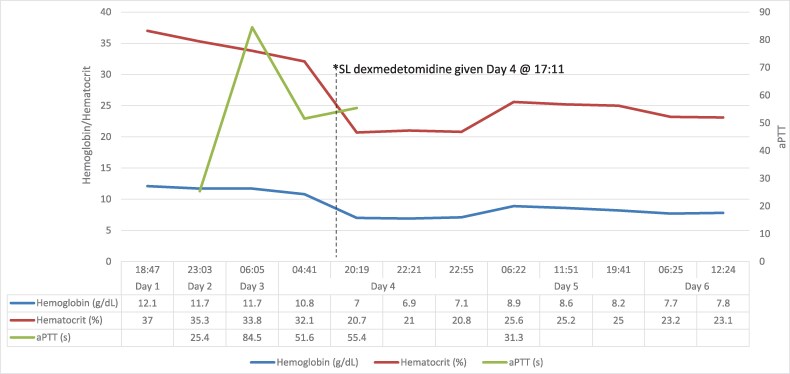
Trends in the patient’s hemoglobin, hematocrit, and activated partial thromboplastin time (aPTT) values in the setting of a heparin infusion and sublingual (SL) dexmedetomidine administration.

On day 4, the patient continued to board in the ED without additional need for calming medications. A repeat electrocardiogram revealed atrial fibrillation with a heart rate of 92 beats per minute, with a prolonged Bazett-corrected QT (QTc) interval (502 milliseconds [ms]). The patient was given two 500-mL crystalloid boluses in the early afternoon (1330 hours and 1530 hours). However, in the late afternoon, the patient again became agitated. Due to the prolonged QTc, a decision was made to avoid additional antipsychotics. With the help of the family’s reassurance, a dose of 120 µg of sublingual dexmedetomidine was given at 1711hours. Within the hour of administration, the patient’s blood pressure decreased from 102/49 to 62/40 mm Hg (Figure 1) and the heart rate declined from 101 to 71 beats per minute. The patient remained responsive to tactile and verbal stimuli. The hypotension was treated with a 500-mL crystalloid bolus, resulting in an improved blood pressure, 103/52 mm Hg. Four hours later, an additional 500-mL bolus was given due to a repeat decrease in blood pressure (66/42 mm Hg). A complete blood count revealed an acute decrease in hemoglobin, from 12.1 g/dL on day 1 to 6.9 g/dL at the end of day 4 (Figure 2), raising the suspicion for acute bleeding and requiring transfusion of 1 unit of blood.

The patient underwent a CT scan of her chest, abdomen, and pelvis, demonstrating a large (9.5 × 9.4 cm) intramuscular hematoma in posterior/medial left thigh, without evidence of arterial extravasation. The patient was transferred to the intensive care unit for close monitoring of blood pressure, monitoring of hematoma expansion, and treatment of NSTEMI. After transfer, the patient experienced another acute decrease in hemoglobin (6.6 g/dL), which required a transfusion of an additional unit of blood.

The patient was ultimately discharged on the eighth day of hospitalization; 4 of those days were spent in the ED. Discharge occurred after her mental status improved and her blood pressure, hemoglobin, and hematoma had remained stable for 36 hours.

### Causality analysis

Using the adverse drug reaction probability scale of Naranjo et al,^3^ we assigned a score of 4 (previous reports, + 1; temporal association, + 2; improvement with fluids, + 1; alternative cause, –1; objective repeated blood pressure measurements, + 1), indicating a possible likelihood that sublingual dexmedetomidine caused the precipitous blood pressure decrease.

### Postmarketing data

A report of this case was submitted to the US Food and Drug Administration (FDA) through the MedWatch adverse event reporting system. To further investigate, we conducted a search of the FDA Adverse Event Reporting System (FAERS) database limited to the period from April 2022 to April 2025. The search was restricted by drug name and route (“dexmedetomidine” and “sublingual”), brand name (“Igalmi”), and Medical Dictionary for Regulatory Activities (MedDRA) preferred terms: “hypotension,” “bradycardia,” and “blood pressure decreased.” Including our case, 5 unique reports were identified: 4 described hypotension and 2 described bradycardia, with some cases including both events. Two case reports described improved hemodynamics following discontinuation of sublingual dexmedetomidine (positive dechallenge), and in 2 cases, symptoms recurred upon re-exposure to the drug (positive rechallenge). In each report, sublingual dexmedetomidine was identified as the primary suspect drug in the causality assessment for hypotension and/or bradycardia. The reported cases were considered as serious, life-threatening, or requiring hospitalization (Table 1).

**Table 1.** Case Reports on Sublingual Dexmedetomidine From FDA Adverse Event Reporting System Database

Case ID	MedDRA preferred term	Dose and form	Patient age/sex/weight	Outcome	Dechallenge	Rechallenge	Role
22115096	Bradycardia	NR	63 y/M/58 kg	Life-threatening	Positive	Positive	Primary suspect
	Hypotension	NR	63 y/M/58 kg	Life-threatening	Positive	Positive	Primary suspect
22260929	Hypotension	SL film	F	Other serious	NA	NR	Primary suspect
23111790	Blood pressure decreased	180 µg, SL film	37 y/F	NR	Unknown	Positive	Primary suspect
23779279	Bradycardia	SL film	NR	Other serious	Positive	Negative	Primary suspect
	Hypotension	SL film	NR	Other serious	Positive	Negative	Primary suspect
23986552^a^	Hypotension	120 µg	88 y/F/58.5 kg	Hospitalization; required intervention	Negative	NA	Primary suspect

Abbreviations: FDA, US Food and Drug Administration; MedDRA, Medical Dictionary for Regulatory Activities; NA, not applicable; NR, not reported; SL, sublingual.

^a^The case reported in this article.

## Discussion

We report a case involving a medically complex octogenarian who presented with NSTEMI requiring anticoagulation and dual antiplatelet therapy along with treatment using oral and parenteral antipsychotics for persistent agitation and delirium—symptoms exacerbated by prolonged boarding in the ED. Following administration of sublingual dexmedetomidine, the patient experienced an abrupt drop in blood pressure that destabilized previously stable hemodynamics and led to escalation of care. While the primary suspected cause of instability was intramuscular hematoma expansion due to therapeutic anticoagulation, dexmedetomidine exacerbated the clinical course by precipitating a dangerous 40-point drop in systolic blood pressure that persisted for a few hours.

We assigned a possible likelihood that sublingual dexmedetomidine contributed to the patient’s hypotension, based on scoring with the Naranjo adverse drug reaction probability scale. Most criteria such as previous reports, temporal association, response to reversal (fluid resuscitation), and objective clinical findings were clearly met. However, the criterion addressing alternative causes presented a challenge. Per the Naranjo scale, the presence of a plausible alternative explanation, such as acute blood loss in this case, requires a score of –1. Although the patient’s hemoglobin declined significantly, her hemodynamics remained relatively stable until the administration of dexmedetomidine, after which she experienced a rapid and profound drop in blood pressure. This temporal pattern strongly suggests a contributory role for dexmedetomidine. Nevertheless, to maintain adherence to the validated scoring structure, we assigned a value of –1 for this item, yielding a total score of 4 and supporting a classification of a possible adverse drug reaction. Of note, the patient did not receive any α_1_ blockers, benzodiazepines, opioids, or other QTc-prolonging medication that could have confounded dexmedetomidine’s clinical effect. This case underscores the limitations of structured causality assessment tools in complex clinical scenarios, where multifactorial contributors may obscure clear attribution.

To our knowledge, this is the first published report of a clinically significant hypotensive event following sublingual dexmedetomidine administration. The drug was approved based on 2 pivotal trials, the SERENITY I and II trials, which evaluated its use for agitation associated with schizophrenia or bipolar I/II disorder.^2,4,5^ In these trials, the average participant age was 46 or 47 years, with only 11 patients over the age of 65; the oldest reported participant was age 71. Hypotension occurred in 5% to 6% of patients, with a mean systolic blood pressure decrease of 18 mm Hg (SD, 16 mm Hg). Assuming a normal distribution, roughly 16% of patients may have experienced a drop of 34 mm Hg or more. In younger, otherwise healthy individuals, many likely hypertensive due to agitation, such a reduction may carry limited clinical significance. However, in older adults with lower baseline systolic pressures, these same drops could have far greater hemodynamic consequences. A recent trial (ClinicalTrials.gov identifier, NCT04251910)^6^ examining sublingual dexmedetomidine in agitation related to dementia (mean participant age, 76 years) used lower doses (30-90 µg) than those in the SERENITY trials (120-180 µg). Although specific blood pressure data were not numerically reported, hypotension was noted in 9% to 25% of patients across treatment arms.

This age-related vulnerability highlights why hypotension remains one of the more clinically concerning adverse effects of sublingual dexmedetomidine. While only 5 postmarketing cases of hypotension/bradycardia were identified in pharmacovigilance data, the FAERS is prone to significant underreporting and does not capture the true incidence of adverse events. Moreover, such databases are not authoritative sources for determining causality, as reports may be incomplete, duplicative, or lack clinical context.^7^ When considered alongside trial data, however, the potential for clinically significant hypotension becomes more apparent. This is particularly relevant in elderly patients, especially those with baseline hypertension, where abrupt reductions in blood pressure may impair myocardial and cerebral perfusion, paralleling the harms previously observed with rapid blood pressure reduction with immediate-release nifedipine.^8^ Of note, the current prescribing information^2^ recommends a reduced dose of 120 µg for patients over 65 years of age; however, despite this dose adjustment, our patient experienced a hypotensive episode.

The pivotal trials supporting the approval of sublingual dexmedetomidine did not identify clinically meaningful changes in the QTc interval. Nonetheless, the prescribing information for sublingual dexmedetomidine states that the drug is associated with concentration-dependent QTc prolongation, with increases of 6-7 ms observed with the 120-µg dose.^2^ In contrast, the package insert for the intravenous formulation does not include a warning related to QTc effects.^1^ According to the website CredibleMeds.org,^9^ dexmedetomidine is listed as a drug that may cause QT prolongation, though it is not currently associated with a known risk of torsades de pointes when used as directed. Published data on intravenous dexmedetomidine and QTc interval prolongation are conflicting, with some studies reporting mild prolongation and others noting either neutral or shortening effects.^10-13^ In our case, the patient developed QTc prolongation in the setting of hypokalemia and coadministration of antipsychotic medications known to affect cardiac repolarization. Although sublingual dexmedetomidine resulted in a marked drop in blood pressure, it did not induce bradycardia or ventricular arrhythmias—including torsades de pointes—even when administered to a patient with a baseline QTc of 502 ms. Unfortunately, no further electrocardiograms were available for review after sublingual dexmedetomidine was given.

Several confounding factors likely contributed to the complexity and deterioration of our patient’s clinical course. Most notably, she experienced a prolonged and arguably unacceptable boarding period in the ED due to hospital overcrowding. Prolonged ED boarding has been linked to numerous adverse outcomes, particularly in older adults. For instance, one study found that critically ill patients aged 65 and older who boarded in the ED for more than 8 hours had longer ICU stays, higher treatment costs, and increased mortality.^14^ Older adults are also especially vulnerable to developing delirium and agitation, both of which have been directly associated with extended ED stays.^15,16^ The ED environment is inherently unfriendly to geriatric patients—often lacking windows, consistent lighting, and quiet spaces for rest—factors that can exacerbate confusion and agitation. Even in our institution, which holds Geriatric Emergency Department Accreditation from the American College of Emergency Physicians, we were unable to prevent the deliriogenic harms of prolonged ED boarding in this medically complex patient. Her agitation—likely exacerbated by both the environment and her underlying medical issues and delirium—required repeated administration of oral and parenteral sedating agents. As intravenous access became limited, the clinical team faced a particularly difficult situation: managing severe agitation in a patient with therapeutic anticoagulation, without reliable IV access, and with the need for repeated intramuscular injections. This route, while necessary, carries known risks—especially in anticoagulated patients—such as localized trauma, bruising, and bleeding. The effect of repeated IM injections in this context contributed to the development of an intramuscular hematoma. These events unfolded against a backdrop of complex medical comorbidities, including recent NSTEMI and a known history of delirium, further amplifying the patient’s vulnerability and the potential for harm.

Our case highlights the complexity of managing delirium and agitation in an elderly, medically fragile patient. Even in the absence of environmental stressors such as ED boarding, selecting an agent that effectively calms without introducing significant risk is a persistent challenge. Benzodiazepines, while sometimes effective for acute agitation, are known to exacerbate delirium and may lead to prolonged cognitive dysfunction, especially in the elderly.^17^ Antipsychotics carry their own risks, including increased mortality in patients with dementia-related psychosis, extrapyramidal symptoms, and QTc prolongation.^18^ In patients with Parkinson’s disease, antidopaminergic properties of many antipsychotics may further worsen motor symptoms, adding yet another layer of complexity to pharmacologic decision-making.

Sublingual dexmedetomidine represents a novel and potentially valuable option for the treatment of agitation. Although its current FDA approval is limited to agitation associated with schizophrenia or bipolar disorder, its mechanism of action suggests broader applicability—similar to the established use of intravenous dexmedetomidine in critical care settings.^19^ Nevertheless, hypotension remains a clinical concern, particularly in older adults with multiple comorbidities. Future studies should focus on identifying the lowest effective sublingual dose that maintains efficacy while minimizing hemodynamic compromise, especially in high-risk populations such as the elderly.

## Conclusion

This case illustrates the potential hemodynamic risks of sublingual dexmedetomidine in a medically complex elderly patient. The patient experienced profound hypotension following sublingual dexmedetomidine administration, contributing to significant clinical deterioration in the setting of prolonged ED boarding, persistent agitation, and an intramuscular hematoma. While sublingual dexmedetomidine offers a novel, noninvasive option for agitation management, its use in older adults with multiple comorbidities warrants careful patient selection, close monitoring, and further investigation to determine optimal dosing strategies that balance efficacy with safety.

## Data Availability

The data underlying this article cannot be shared publicly due to the privacy of the individual described in this case report.

## Additional information

The authors provided the following information regarding their contributions to this work: Z.C., P.T., and M.A. conceived the report and drafted the initial manuscript. C.H. and B.M. significantly contributed to manuscript editing and content. Z.C. and P.T. take responsibility for the paper as a whole.
